# **Sialic acid-responsive** ***Parabacteroides*** **is linked to gut barrier integrity in older adults**

**DOI:** 10.1080/19490976.2026.2627093

**Published:** 2026-02-10

**Authors:** Shin Fujiwara, Jonguk Park, Mariko Takeda, Takumi Miyatake, Yoshie Saito, Seiya Makino, Yun-Gi Kim

**Affiliations:** a Wellness Science Labs, Meiji Holdings Co., Ltd., Tokyo, Japan; b R&D Division, Meiji Co., Ltd., Tokyo, Japan; c Department of Microbiology, School of Pharmacy, Kitasato University, Tokyo, Japan

**Keywords:** Inflammaging, intestinal barrier, microbiota, *Parabacteroides*, sialic acid, mucin

## Abstract

Aging is frequently accompanied by inflammaging—a chronic, low-grade inflammatory state that contributes to functional decline and disease risk. Disruption of the intestinal barrier is increasingly being recognized as a key driver of inflammaging; however, its relationship with the gut microbiota in older adults remains poorly understood. Here, we demonstrate a significant association of intestinal barrier dysfunction markers with systemic inflammatory markers using a cross-sectional study in this population. Notably, the genus *Parabacteroides* showed a strong negative association with barrier dysfunction. *In vitro* assays showed that three *Parabacteroides* lineages predominant in older adults, including *P. merdae*, enhanced the intestinal barrier integrity in a viability-dependent manner. Fecal sialic acid (Neu5Ac) levels were positively correlated with the abundance of *Parabacteroides*. Mediation analysis further indicated that *Parabacteroides* significantly mediated the association between fecal sialic acid and intestinal barrier markers. Culture experiments showed that both sialic acid and mucin, which is rich in terminal sialyl residues, promoted *Parabacteroides* growth. Transcriptomic analysis of *P. merdae* cultured with sialic acid revealed upregulation of genes for sialidases, transporters, and enzymes, consistent with sialic acid catabolism and transport, suggesting utilization of mucin-derived sialic acid. Together, these findings indicate that in older adults, *Parabacteroides* is linked to the intestinal barrier integrity and responds to mucin-associated sialic acid, supporting a model wherein host-derived glycans foster barrier-protective microbes to promote healthy aging. The study findings provide avenues for devising strategies for maintaining the intestinal barrier integrity and reducing age-related inflammation, which may ultimately contribute to the prevention of inflammaging.

## Graphical abstract

**Figure:**
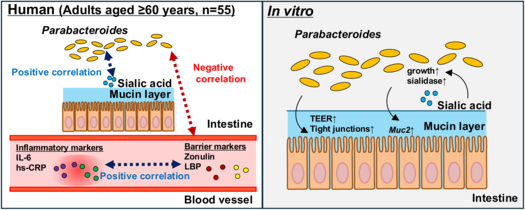

## Introduction

Aging is accompanied by a chronic, low-grade inflammatory state known as inflammaging,^1^ which increases the risk of age-associated pathologies, including cardiovascular disease, chronic kidney disease, diabetes mellitus, sarcopenia, depression, dementia, and cancer.^2^^,^^3^ Although inflammaging is driven by multiple factors, dysfunction of the intestinal barrier is increasingly being recognized to play a central role in its development.^2^^,^^4^^,^^5^

The intestinal barrier provides a critical interface between the host and external environment.^6^ Disruption of this barrier—often referred to as “leaky gut”—increases intestinal permeability, allowing microbial components such as lipopolysaccharides (LPS) to enter the systemic circulation and trigger inflammation.^7^ Among the available biomarkers of intestinal barrier function, serum zonulin, a physiological modulator of tight junctions, and lipopolysaccharide-binding protein (LBP), a protein that binds LPS and facilitates immune recognition, are widely used. The levels of both these markers increase with age and correlate positively with the levels of proinflammatory cytokines,^8^^,^^9^ underscoring their utility in assessing the links between age-related barrier dysfunction and chronic low-grade inflammation.

The gut microbiota is closely related to the intestinal barrier function. In conditions, such as inflammatory bowel disease^10^^,^^11^ and type 2 diabetes,^10^^,^^12^^,^^13^ barrier impairment is accompanied by microbial dysbiosis, characterized by reduced abundance of beneficial taxa, including *Bifidobacterium*, *Faecalibacterium*, and *Akkermansia*, in the gut. These bacteria enhance the barrier integrity via distinct mechanisms; for example, *Bifidobacterium* does so via acetate production,^14^ *F. prausnitzii* via butyrate,^15^^,^^16^ and *A. muciniphila* via acetate, propionate, and the membrane protein Amuc_1100.^15^^,^^17-19^ Reduced abundance of these taxa has been linked to compromised barrier function. However, therapeutic interventions, and the disease itself, can markedly alter the gut microbiota composition,^20^^-^^22^ making the identification of bacterial taxa that primarily support barrier integrity in the general population difficult.

Although zonulin and LBP are associated with inflammatory markers in various clinical contexts, studies focusing on these markers in relation to the gut microbiota and microbial metabolites in older adults remain scarce. For preventing inflammaging, identification of microbial taxa that contribute to the maintenance of the intestinal barrier under physiological aging is important.

To bridge the abovementioned gap in knowledge, we investigated the association of the intestinal barrier function with gut microbiota composition and the related fecal metabolites in older adults. We first examined the relationships between barrier function markers and phenotypic metadata, including systemic inflammatory markers. Next, we identified bacterial taxa associated with barrier integrity and evaluated the effects of candidate bacteria on the barrier function *in vitro*. Finally, we explored potential microbial metabolites linked to these taxa, aiming to gain mechanistic insights into microbiota–barrier interactions in aging.

## Materials and methods

### Study design

A cross-sectional study was conducted between December 2019 and February 2020. Participants were eligible for inclusion if they were aged over 60 years at the time of consent, regardless of gender. All participants were fully apprised of the purpose of the study and procedures involved, demonstrated the capacity to provide informed consent, and voluntarily agreed to participate by signing a written informed consent form. The individuals who were receiving treatment for serious gastrointestinal diseases, had undergone surgical removal of digestive organs, had taken antibiotics, metformin, or proton pump inhibitors within one month prior to obtaining consent were excluded from the study.

This study was approved by the Institutional Review Board of Chiyoda Paramedical Care Clinic (approval number: 19122002) and the Meiji Institutional Review Board (approval number: 180) and was conducted in accordance with the Declaration of Helsinki. All participants provided written informed consent. This study was registered at the University Hospital Medical Information Network Clinical Trials Registry (trial ID: UMIN000039151).

### Baseline assessments

Baseline assessments included questionnaire-based evaluations and physical measurements. The participants completed a comprehensive, self-administered questionnaire to collect information on health-related habits and conditions. Sleep quality and daytime sleepiness were evaluated using the Pittsburgh sleep quality index (PSQI)^23^ and the Epworth sleepiness scale (ESS),^24^ respectively. Fatigue was assessed using the Chalder fatigue scale (CFS),^25^ and gastrointestinal symptoms were evaluated employing the gastrointestinal symptom rating scale (GSRS).^26^ Dietary habits were assessed using the brief-type self-administered diet history questionnaire (BDHQ).^27^ Frailty status was evaluated using the Japanese version of the Cardiovascular Health Study (J-CHS) criteria.^28^

### Anthropometric measurements

Body composition, including skeletal muscle mass and body fat percentage, was assessed using InBody430 (InBody Japan Inc., Tokyo, Japan). Walking speed was evaluated by measuring the time taken to walk a distance of 4.6 m. Grip strength was measured three times on each hand using a handheld dynamometer, and the maximum value was used for analysis.

### Blood analysis

The participants were instructed to fast after 11:00 p.m., a day prior to blood sample collection. Blood was collected and aliquoted into three types of collection tubes: whole blood was collected in a tube containing EDTA-2K for hematological analysis; for plasma preparation, blood was drawn into a tube containing sodium fluoride and centrifuged at 1,730 × *g*, for 10 min at 4 °C; and for serum preparation, blood was placed in a tube containing a separation gel and centrifuged at 1,730 × *g*, for 10 min at 4 °C. Each tube was stored under appropriate temperature conditions until analysis. General biochemical tests, immunological assays, and hematological analyses were performed using whole blood, serum, or plasma, depending on the test. All analyses were conducted by SRL, Inc. (Tokyo, Japan).

To assess the intestinal barrier function, serum LBP levels were measured using the Human LBP ELIZA Kit (Hycult Biotech, Uden, The Netherlands) and zonulin levels were determined using the IDK Zonulin ELIZA Kit (Immundiagnostik AG, Bensheim, Germany), according to the manufacturers’ instructions.

### Fecal sample analysis

Fecal samples collected by the participants at their homes were refrigerated for up to 1 day and then frozen in a −80 °C freezer until analysis.

The content of fecal mucin and immunoglobulin A (IgA) was analyzed by Cosmo Bio Co., Ltd. (Tokyo, Japan). Briefly, fecal mucin content was determined using a Fecal Mucin assay kit (Cosmo Bio Co., Ltd., Tokyo, Japan) and fecal IgA content was determined via ELIZA using a Human IgA ELIZA Quantitation Set (Bethyl Laboratories, Inc., Montgomery, TX, USA).

Fecal samples were suspended in phosphate-buffered saline (PBS) at a ratio of 1:5 (w/v), followed by the addition of 1/5 volume of 50 mM NaOH. After thorough mixing, the samples were centrifuged at 13,000 rpm for 10 min at 4 °C. The supernatant was collected and mixed with an equal volume of chloroform, followed by another round of centrifugation at 3,000 rpm for 10 min at 4 °C. The supernatant was then filtered through 0.2 µm filter vials and used for analyzing short-chain fatty acids (SCFAs).

High-performance liquid chromatography (HPLC) was performed to quantify SCFAs, including acetate, propionate, and butyrate, using two serially connected ICSep ICE ORH-801 guard columns (Concise Separations, San Jose, CA, USA). The mobile phase consisted of 7.5 mM *p*-toluene sulfonic acid (FUJIFILM Wako Pure Chemical Corporation, Osaka, Japan) dissolved in distilled water, whereas the reaction phase contained 7.5 mM *p*-toluene sulfonic acid, 150 µM EDTA-2Na (Dojindo, Kumamoto, Japan), and 30 mM Bis-Tris (Dojindo) in distilled water. The flow rate was maintained at 0.5 mL/min, and the column temperature was set to 55 °C. SCFAs were detected using a conductivity detector CDD-10AVP (Shimadzu Corporation, Kyoto, Japan). Sodium acetate trihydrate, sodium propionate, and sodium butyrate were used as standards to calculate the concentrations of SCFAs.

### DNA extraction

Fecal DNA was extracted as described previously.^29^ Briefly, approximately 20 mg of fecal sample was transferred into a 2.0 mL tube containing 0.3 g of 0.1 mm zirconia beads (BioSpec Products, Inc., Bartlesville, OK, USA) and homogenized using a FastPrep system (MP Biomedicals, LLC, Irvine, CA, USA). DNA was extracted using the QIAamp Fast DNA Stool Mini Kit (QIAGEN, Hilden, Germany). DNA integrity was assessed via electrophoresis on a 1% agarose gel, and DNA concentration was measured using a Qubit fluorometer (Thermo Fisher Scientific Inc., Waltham, MA, USA).

### 16S rRNA gene sequencing and microbiota analysis

Polymerase chain reaction (PCR) for amplification of the 16S rRNA gene was conducted using universal primers targeting the V3-V4 region of the bacterial 16S rRNA gene. The amplified DNA was purified using AMPure XP beads (Beckman Colter Life Sciences, Indianapolis, IN, USA), and adapters were added via PCR using a Nextera XT index kit (Illumina, Inc., San Diego, CA, USA). Sequencing was performed using a MiSeq System (Illumina, Inc.) with a MiSeq Reagent Kit V3 (600-cycle). Sequencing data were processed and analyzed using QIIME2 version 2020.11.^30^ The Cutadapt plugin in QIIME2 was used to trim the primer regions from the raw sequences. Sequences without primer regions were denoised using the DADA2 algorithm.^31^ Total reads for each sample were rarefied to 10,000 by random sampling to ensure comparability across samples and consistency with downstream diversity and correlation analyses. For taxonomic classification, a pretrained Naive Bayes classifier based on the SILVA database (version 132)^32^ was used to assign taxonomy to each amplicon sequence variant (ASV). α diversity was calculated using the QIIME2 software, and β diversity was calculated based on the relative abundance of each ASV using the Bray–Curtis dissimilarity metric using the vegan package. The relative abundance of each genus with an average relative abundance of at least 1% was analyzed.

ASVs assigned to the genus *Parabacteroides* were extracted, with those representing at least 1% of the total relative abundance of *Parabacteroides*-assigned ASVs being included. These ASVs were assigned to the most closely related species by performing a BLASTn homology search against the NCBI 16S rRNA sequence database with the highest similarity.

### Metabolomics analysis

Metabolomics analysis was performed using the Basic Scan package from Human Metabolome Technologies, Inc. (Turuoka, Japan), utilizing the capillary electrophoresis–time-of-flight–mass spectrometry (CE-TOFMS) technology. Fecal metabolites were extracted by thoroughly shaking the sample with sterilized water containing the internal standard. The resulting mixture was then centrifuged, and the aqueous supernatant was filtered using centrifugal filtration according to the manufacturer’s instructions. The peaks identified CE-TOFMS were processed with an automatic integration software (MasterHands, Keio University, Japan) to extract peak data, including the mass-to-charge (*m/z*) values, migration times (MT), and peak areas. These peaks were annotated by matching them to potential metabolites in the HMT metabolite database, using their MTs in CE and *m/z* values from TOF-MS. The peak annotation tolerance was set at ±0.5 min for MT and ±10 ppm for *m/z*. For correlation and multivariate analyses, only fecal metabolites with non-zero values across all samples were included.

### Cell culture

For assessing the intestinal barrier function, human colorectal epithelial Caco-2 cells were purchased from the European Collection of Authenticated Cell Cultures and cultured in a minimum essential medium (Thermo Fisher Scientific Inc.) supplemented with 10% (v/v) fetal bovine serum (Biowest, Nuaillé, France), 1% (v/v) MEM non-essential amino acid solution (Thermo Fisher Scientific Inc.), 100 U/mL penicillin, and 100 µg/mL streptomycin (Thermo Fisher Scientific Inc.). The culture was maintained at 37 °C in a 5% CO_2_ atmosphere. The medium without penicillin and streptomycin was used for the coculture of *Parabacteroides* and Caco-2 cells. For evaluating *Muc2* expression, human colorectal LS174T cells, purchased from the American Type Culture Collection, were cultured in MEM (Thermo Fisher Scientific Inc.) supplemented with 10% (v/v) fetal bovine serum (Biowest), 1% (v/v) L-glutamine, 100 U/mL penicillin, and 100 µg/mL streptomycin (Thermo Fisher Scientific Inc.). The culture was maintained at 37 °C in a 5% CO_2_ atmosphere. The medium without penicillin and streptomycin was used for the coculture of *Parabacteroides* and LS174T cells.

### Bacterial treatment

*P. merdae* JCM9497^T^, *P. distasonis* JCM5825^T^, and *P. johnsonii* JCM13406^T^ were purchased from the Japan Collection of Microorganisms (JCM, RIKEN BioResource Research Center, Ibaraki, Japan). These strains were recovered and maintained in Gifu Anaerobic Medium (GAM) broth (Nissui Pharmaceutical Co., Ltd., Tokyo, Japan) in an anaerobic chamber (Coy Laboratory Products, Inc., Grass Lake, MI, USA) with anaerobic conditions (80% N_2_, 10% CO_2_, 10% H_2_) at 37 °C. The bacterial culture was centrifuged at 6,000 × *g* for 3 min, washed twice with PBS (pH 7.4), and then resuspended in cell culture medium without penicillin and streptomycin to obtain bacterial suspension with an optical density at 650 nm (OD_650_) of 10.0. Heat-killed (HK) bacteria were obtained by incubating the bacterial suspension at 90 °C for 15 min.

### Transepithelial electrical resistance (TEER) measurements

To assess the intestinal barrier function, Caco-2 cells were seeded on 12-well Transwell inserts (Corning Inc., Corning, NY, USA) at a concentration of 1.0 × 10^5^ cells/well and cultivated for 14 days to form a monolayer. The medium was changed every two or three days. The TEER value was measured using a MILLICELL-ERS voltohmmeter system (Millipore–Sigma, Burlington, MA, USA); only values of 500 Ώ per well were used. Thereafter, bacterial suspension or culture medium (as control) was added to the apical side to get an OD_650_ of 0.05 or 0.1 and incubated at 37 °C for 18 h in a 5% CO_2_ atmosphere. The TEER value was measured at 0 and 18 h. The resulting data are presented as a ratio.

Ratio = (TEER treatment 18 h—TEER treatment 0 h)/(TEER control 18 h—TEER control 0 h).

### Coculture of LS174T cells with bacteria

LS174T cells were seeded at a density of 1.0 × 10^5^ cells/well in 24-well plates. After 2 days of culture, the culture medium was removed, and the cells were replenished with the medium without penicillin and streptomycin. Subsequently, bacterial suspension was added to achieve an OD_650_ of 0.1. The cells were then incubated for 24 h at 37 °C in a 5% CO_2_ atmosphere.

### RNA isolation and gene expression analysis

Total RNA from Caco-2 and LS174T cells was extracted using a Maxwell RSC48 automatic nucleic acid extractor (Promega Corporation, Madison, WI, USA) and a Maxwell RSC Simply RNA Cells Kit (Promega Corporation), following the manufacturer’s instructions. Complementary DNA was synthesized from 1 μg of total RNA using a PrimeScript RT Master Mix (Takara Bio Inc., Kusatsu, Japan), and real-time PCR was performed using a QuantStudio 3 Real-Time PCR System (Thermo Fisher Scientific Inc.) and TBGreen Premix Ex Taq (Takara Bio Inc.), according to the manufacturers’ instructions. The nucleotide sequences of the primers used in this study are listed in Table S1. Quantitative comparisons were performed using the 2^−ΔΔCT^ method.^33^ Data were normalized against the values for *GAPDH* and the results are presented as relative expression against control.

### Growth experiments

*P. merdae* JCM9497^T^, *P. distasonis* JCM5825^T^, and *P. johnsonii* JCM13406^T^ were recovered and maintained in GAM broth (Nissui Pharmaceutical Co., Ltd.) in an anaerobic chamber (Coy Laboratory Products, Inc.) under anaerobic conditions at 37 °C. For growth experiments, YCFA medium (no. 1130, https://www.jcm.riken.jp/cgi-bin/jcm/jcm_grmd?GRMD=1130) without sugars (pH 7.45) was used as the base medium supplemented with 10 mM *N*-acetylneuraminic acid (Neu5Ac; FUJIFILM Wako Pure Chemical Corporation), 1% (w/v) mucin (FUJIFILM Wako Pure Chemical Corporation) or sterile water as a control. The bacterial culture was added to this medium at 1% (v/v) and incubated for 24 or 48 h under anaerobic conditions at 37 °C. The OD_650_ of bacterial culture was determined at each time point.

### RNA extraction from *P. merdae*, sequencing, and transcriptome analysis

*P. merdae* JCM9497^T^ was cultured in GAM broth (Nissui Pharmaceutical Co., Ltd.) under anaerobic conditions. The bacterial culture was added to YCFA medium without sugars, supplemented with 10 mM Neu5Ac (FUJIFILM Wako Pure Chemical Corporation), and incubated for 8 h under anaerobic conditions at 37 °C. The culture was then centrifuged at 10,000 rpm for 5 min at 4 °C and the supernatant was removed. The bacterial pellets were flash frozen in liquid nitrogen. Bacterial cells were first lysed in LETS buffer (100 mM LiCl, 10 mM EDTA, 10 mM Tris-HCl; pH 7.4, 1% SDS). The suspension was transferred to a Lysing Matrix B tube containing 500 µL of phenol-chloroform-isoamyl alcohol (PCI, 25:24:1). The cells were lysed using a FastPrep system (MP Biomedicals, LLC, Irvine, CA, USA) at a speed of 6.5 for 45 s, twice, with cooling on ice between cycles, and the lysate was centrifuged at 7,000 rpm for 10 min at 4 °C. The aqueous phase was transferred to a MaXtract High Density tube precentrifuged at 15,000 rpm for 1 min, and 500 µL of PCI was added to it. The mixture was gently inverted five times and centrifuged at 15,000 rpm for 10 min at 4 °C. The upper aqueous phase was transferred to a new 2 mL tube and RNA was precipitated by adding 1/10 volume of 1 M LiCl and 2.5 volumes of ice-cold 100% ethanol, followed by vortexing for 20 s. The sample was centrifuged at 15,000 rpm for 15 min at 4 °C and washed with 200 µL of 70% ethanol. Subsequently, RNA was purified using the NucleoSpin RNA kit (Macherey-Nagel GmbH & Co. KG, Düren, Germany) following the manufacturer’s instructions. RNA integrity was assessed using an Agilent 2100 Bioanalyzer (Agilent Technologies, Inc., Santa Clara, CA, USA), and samples with an RNA integrity number >9.0 were used for further analysis.

Ribosomal RNA was depleted using the MicrobExpress Bacterial mRNA Enrichment Kit (Thermo Fisher Scientific Inc.) according to the manufacturer’s instructions. RNA-Seq libraries were prepared using the TruSeq Stranded mRNA Library Prep Kit (Illumina, Inc.) following the manufacturer’s instructions. First-strand cDNA synthesis was performed using SuperScript II Reverse Transcriptase (Thermo Fisher Scientific Inc.). Unique dual indices were assigned to each sample using the IDT for Illumina TruSeq RNA UD Index Set (Illumina, Inc.). Library quality and fragment size distribution were assessed using an Agilent 2100 Bioanalyzer (Agilent Technologies, Inc.). The concentration of library was determined using a Qubit Fluorometer (Thermo Fisher Scientific Inc.). Sequencing was performed using a MiSeq System (Illumina, Inc.) with the MiSeq Reagent kit V3 (150-cycle).

The adapter sequences and low-quality bases were trimmed using fastp v0.20.1. The reads were mapped to the *P. merdae* reference genome (accession no. GCF_025151215.1) using HISAT2 v2.1.0 with default parameters. Sorted BAM files were generated using SAMtools v1.10. The expression levels of genes were determined using StringTie v2.1.2. Differentially expressed genes were identified using the DESeq2 package with a threshold of |log_2_ fold change| > 2.0 and *p* value < 0.05.

### Statistical analysis

All statistical analyses were conducted using the R software, version 4.3.2 (R Foundation, Viennam, Austria). Correlation analyses were performed by calculating Spearman’s correlation coefficients using the stats package. Multiple linear regression analyses were performed to further evaluate associations identified by Spearman’s correlation analyses. Age, sex, and body mass index (BMI) were included as confounders in all models. For analyses examining associations between gut microbial taxa and intestinal barrier markers, two multiple linear regression models were specified: Model 1 was adjusted for age, sex, and BMI, and Model 2 was further adjusted for GSRS score and total dietary fiber intake (g/1000 kcal/day).

Associations between intestinal barrier markers and gut microbial taxa, as well as between selected bacterial taxa and fecal metabolome, were examined using redundancy analysis (RDA) with stepwise variable selection based on adjusted R². When multiple variables were identified, they were included as explanatory variables in multiple linear regression models. In addition to the multivariate analyses, differential abundance analysis was performed using DESeq2 on the raw count data, with size factor normalization, to examine associations between intestinal barrier markers and microbial taxa. Prior to analysis, taxa with low counts (total counts < 50,000 across all samples) and low prevalence ( < 50% of samples) were filtered. Mediation analyses were conducted using the mediation package.

For comparisons between two groups, either Student’s *t*-test or the Wilcoxon rank-sum test was applied using the rstatix package. For comparisons involving three or more groups, Dunnett’s multiple comparisons test or Dunn’s test following a Kruskal–Wallis test was performed using the stats, multcomp, and rstatix packages. A two-sided *p* value < 0.05 was considered statistically significant. For Spearman’s correlation analyses, *p* values were adjusted for multiple comparisons using the FDR correction (Benjamini–Hochberg), and both raw and FDR-adjusted *p* values are reported in the Supplementary Tables.

## Results

### Participant characteristics

Of the 56 recruited participants, one was excluded because of the inflammatory marker levels exceeding the normal reference range. Analyses were, therefore, conducted on 55 participants. Baseline characteristics are summarized in Table 1.

**Table 1.** Baseline characteristics of the participants.

Characteristic	*N* = 55^a^
Age, y	69.2 (4.9)
**Sex**	
Female	28 (51)
Male	27 (49)
Height, cm	161 (8)
Weight, kg	58 (10)
BMI, kg/m^2^	22.5 (3.4)
Exercise, *n* (%)	34 (62)
Alcohol intake, *n* (%)	26 (47)
Smoking status, *n* (%)	4 (7.3)
Lean mass, kg	42 (8)
SMI, kg/m2	6.53 (0.96)
Walking speed, second	3.18 (0.43)
Grip strength, kg	31 (8)
PSQI	4.04 (2.44)
ESS	5.3 (4.2)
CFS	11.3 (6.4)
GSRS	1.44 (0.41)
**J-CHS**	
0	32 (58)
1	18 (33)
2	4 (7.3)
3	1 (1.8)
Energy intake, kcal/day	1,856 (556)
Dietary protein intake, g/1000kcal/day	41 (9)
Dietary fat intake, g/1000kcal/day	32.3 (6.2)
Dietary carbohydrate intake, g/1000kcal/day	129 (21)
Dietary fiber intake, g/1000kcal/day	7.54 (2.17)
IL-6, pg/mL	1.63 (1.19)
hs-CRP, ng/mL	1,175 (2,555)
Leukocyte, count/μL	5,529 (1,138)
Total protein, g/dL	7.32 (0.39)
Albumin, g/dL	4.37 (0.27)
Urea nitrogen, mg/dL	14.8 (4.0)
Creatinine, mg/dL	0.74 (0.16)
Uric acid, mg/dL	5.18 (1.28)
AST, U/L	23.6 (6.2)
ALT, U/L	21 (12)
*γ*-GTP, U/L	25 (17)
Total cholesterol, mg/dL	232 (36)
Triglyceride, mg/dL	94 (51)
LDL-cholesterol, mg/dL	141 (34)
HDL-cholesterol, mg/dL	73 (18)
Blood glucose, mg/dL	91 (8)
HbA1c, %	5.56 (0.32)
IgA, μg/g	308 (342)
Mucin, μg/g	146 (99)
**Bristol scale**	
1	2 (3.6)
2	1 (1.8)
4	24 (44)
5	21 (38)
6	6 (11)
7	1 (1.8)
**Bristo scalel color**	
1	2 (3.6)
2	34 (62)
3	15 (27)
4	4 (7.3)
defecation frequency, times/week	7.05 (2.91)
defecation amount, count (Equivalent to one medium-sized egg)	2.79 (1.52)

a Mean (SD); n (%).

### Intestinal barrier dysfunction is associated with systemic inflammation in older adults

**Figure 1.**
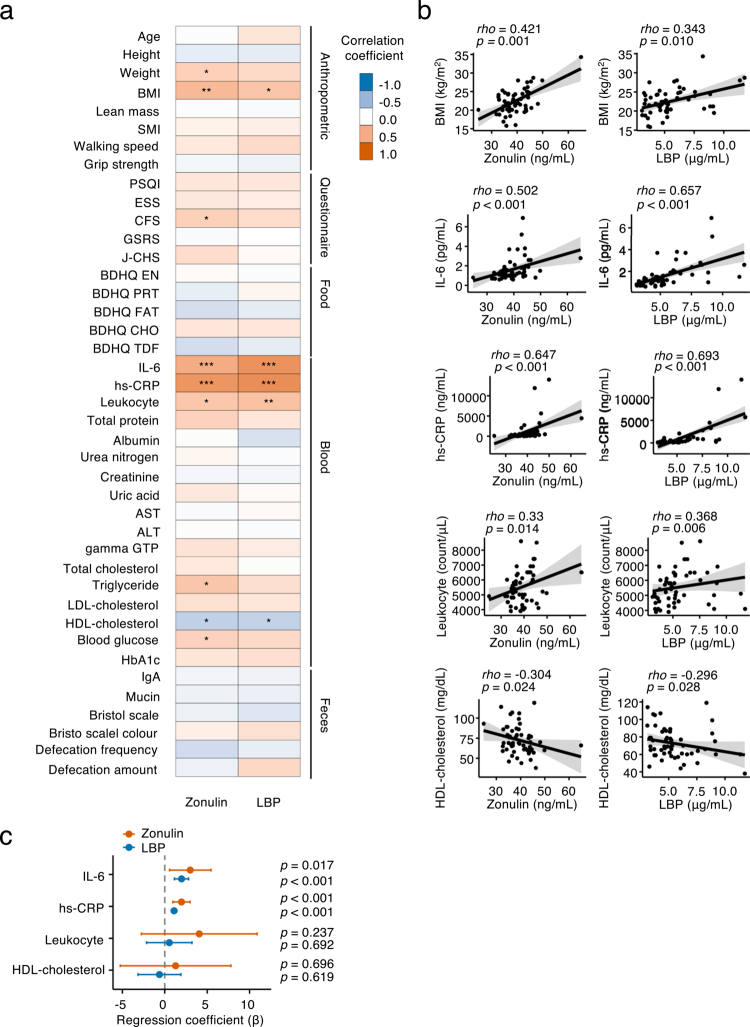
Correlation between phenotypic metadata and intestinal barrier markers (*n* = 55). (a) Spearman’s correlation analysis of zonulin and lipopolysaccharide-binding protein (LBP) levels with continuous variables from phenotypic metadata. (b) Scatter plots of phenotypic metadata correlated with zonulin and LBP levels. (c) Multiple linear regression analysis of zonulin and LBP levels with log-transformed interleukin-6 (IL-6), high-sensitivity C-reactive protein (hs-CRP), leukocyte count, and high-density lipoprotein (HDL)-cholesterol adjusted for age, sex, and body mass index (BMI). Statistical significance was assessed using Spearman’s correlation analysis (a and b) and multiple linear regression analysis (c). *p < 0.05; **p < 0.01; ***p < 0.001. SMI, skeletal muscle mass index; PSQI, pittsburgh sleep quality index; ESS, epworth sleepiness scale; CFS, Chalder fatigue scale; GSRS, gastrointestinal symptom rating scale; J-CHS, Japanese version of the cardiovascular health study; BDHQ EN, brief-type self-administered diet history questionnaire (BDHQ) energy intake (kcal/day); BDHQ PRT, BDHQ protein (g/1000 kcal/day); BDHQ FAT, BDHQ fat (g/1000 kcal/day); BDHQ CHO, BDHQ carbohydrate (g/1000 kcal/day); BDHQ TDF, BDHQ total dietary fiber (g/1000 kcal/day); AST, aspartate transaminase; ALT, alanine transaminase; HbA1c, hemoglobin A1c; IgA, immunoglobulin A.

Assessment of the relationship between the two intestinal barrier markers—zonulin and LBP—revealed a significant positive correlation (Figure S1a). This finding suggests that these markers capture related aspects of the barrier integrity. Spearman’s correlation analysis showed that both zonulin and LBP levels were positively correlated with body mass index (BMI), levels of interleukin-6 (IL-6) and high-sensitivity C-reactive protein (hs-CRP), and leukocyte count, and negatively correlated with the levels of high-density lipoprotein (HDL) cholesterol (Figure 1a, b; Table S2). Zonulin levels were positively associated with body weight, CFS, triglyceride, and blood glucose. Multiple linear regression analyses adjusted for age, sex, and BMI showed IL-6 and hs-CRP were the only variables that were significantly associated with both zonulin and LBP levels (Figure 1c and Figure S1b). For zonulin, IL-6 (*β* = 3.004; 95% CI: 0.560 to 5.449; R² = 0.446) and hs-CRP (*β* = 1.971; 95% CI: 0.958 to 2.983; R² = 0.524) showed significant associations. For LBP, IL-6 (*β* = 1.978; 95% CI: 1.145 to 2.810; R² = 0.453) and hs-CRP (*β* = 1.094; 95% CI: 0.770 to 1.417; R² = 0.585) were also significantly associated. The levels of barrier markers were then compared across categorical variables, including sex, regular exercise habit, alcohol intake habit, and current smoking status, but no significant differences were detected (Figure S1c). Taken together, these results indicate that in older adults, higher levels of intestinal barrier dysfunction markers—as reflected by increased zonulin and LBP levels—are specifically and consistently linked to systemic inflammation, particularly elevated IL-6 and hs-CRP concentrations.

### *Parabacteroides* is the only bacterial genus consistently negatively associated with intestinal barrier markers

**Figure 2.**
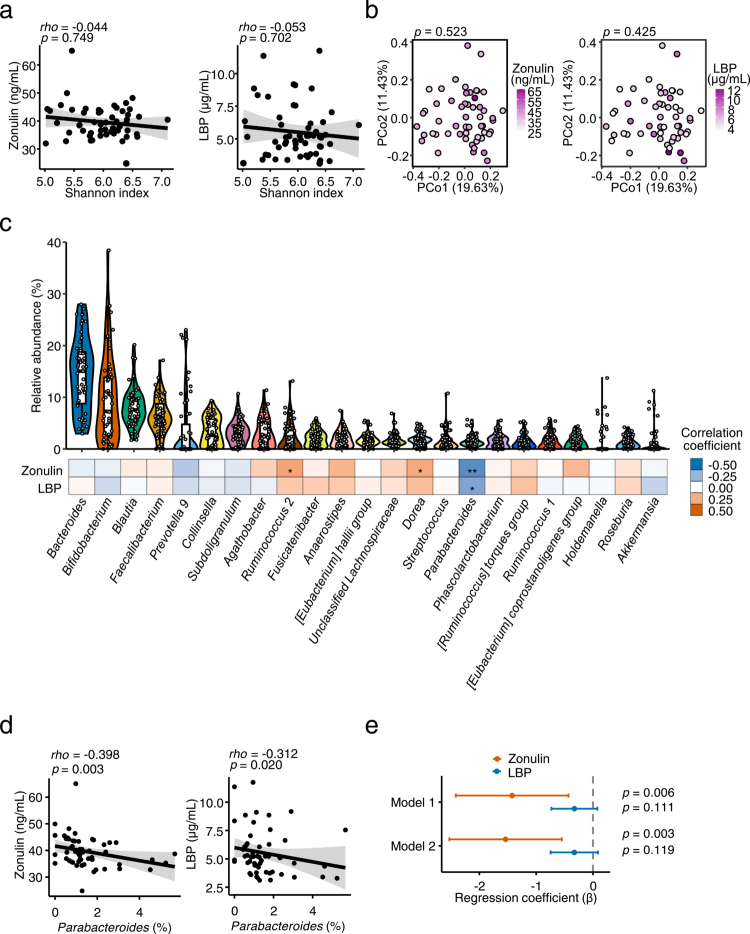
Association between intestinal barrier markers and the gut microbiota (*n* = 55). (a) Spearman’s correlation analysis between the Shannon index and zonulin or lipopolysaccharide-binding protein (LBP) levels. (b) Principal coordinate analysis (PCoA) based on Bray–Curtis dissimilarity of genus-level relative abundance data. Associations between the intestinal barrier markers and microbial community structure was evaluated using the envfit function in vegan package. (c) Violin plots showing the relative abundance of gut microbial taxa, with boxplots and individual data points. The heatmap indicates Spearman’s correlation coefficients with zonulin and LBP levels. Only genera with an average relative abundance of at least 1% were included. (d) Scatter plots showing correlations between *Parabacteroides* abundance and zonulin and LBP levels. (e) Multiple linear regression analysis of zonulin and LBP levels with *Parabacteroides* abundance. Model 1 was adjusted for age, sex, and body mass index (BMI). Model 2 was further adjusted for gastrointestinal symptom rating scale (GSRS) score and total dietary fiber intake (g/1000 kcal/day). Statistical significance was assessed using Spearman’s correlation analysis (a, c, d), envfit function in vegan package (b), and multiple linear regression analysis (e). *p < 0.05; **p < 0.01.

To identify microbial diversity metrics associated with the intestinal barrier function, α diversity was first assessed using the Shannon index. Neither zonulin nor LBP levels showed a significant correlation with α diversity (Figure 2a). β diversity, evaluated using the Bray–Curtis dissimilarity, was also not significantly associated with either marker (Figure 2b). To identify specific gut microbiota taxa associated with the intestinal barrier function, each taxa at the genus level was assessed. *Parabacteroides* correlated significantly and negatively with both the markers (Figure 2c, d; Table S3). Multiple linear regression analyses showed the inverse association between *Parabacteroides* abundance and zonulin levels to be significant after adjusting for potential confounders (Model 1: *β* = −1.423, 95% CI −2.416 to −0.430, R² = 0.467; Model 2: *β* = −1.539, 95% CI −2.529 to −0.548, R² = 0.501; Figure 2e). To further evaluate the robustness of this association, we conducted multivariate analyses using RDA with stepwise variable selection and differential abundance testing via DESeq2. RDA identified *Parabacteroides* as the only genus significantly associated with zonulin (Figure S2a). Furthermore, this was corroborated by the DESeq2 analysis, which identified *Parabacteroides* as the sole genus negatively associated with zonulin (Figure S2b). Several taxa, namely *Bifidobacterium*, *Faecalibacterium*, and *Akkermansia,* previously reported to enhance the barrier integrity in disease contexts,^10^^-^^13^ showed no significant association with zonulin and LBP levels. We also examined fecal SCFAs in relation to barrier markers. Among acetate, propionate, and butyrate, only butyrate was positively associated with zonulin and LBP levels (Figure S3). Taken together, these results identified *Parabacteroides* as the only bacterial genus consistently and negatively associated with the barrier marker in older adults, rather than the taxa previously reported to enhance barrier function via SCFAs production, indicating a robust link between this taxon and the intestinal barrier integrity.

### Three predominant *Parabacteroides* lineages enhance the intestinal barrier integrity *in vitro*

**Figure 3.**
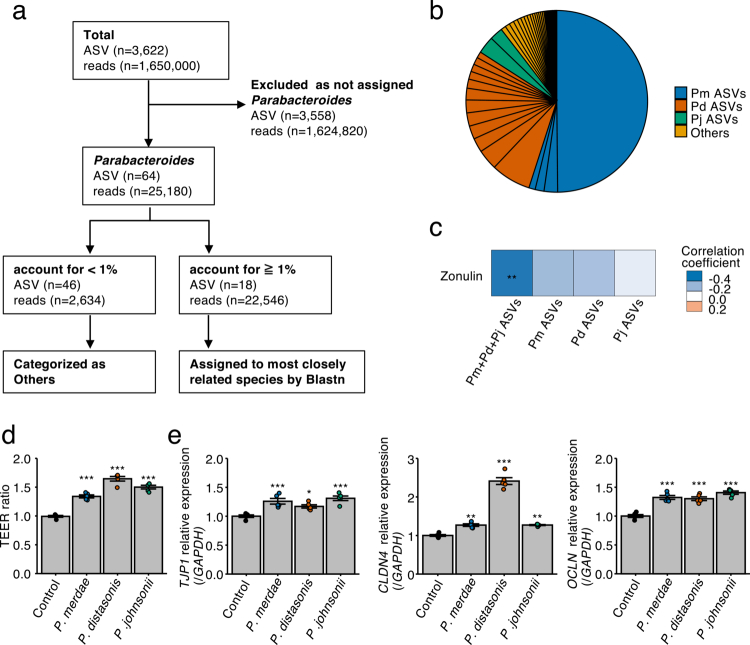
Effects of *Parabacteroides* spp. on the intestinal barrier function in Caco-2 cells. (a) Flowchart illustrating the extraction of amplicon sequence variants (ASVs) assigned to the genus *Parabacteroides*. (b) Pi chart showing the results of BLAST search for *Parabacteroides*-assigned ASVs. Each delimiter represents one ASV. (c) Heatmap indicating Spearman’s correlation coefficients between zonulin levels and the abundance of each *Parabacteroides* lineage or their combined abundance. (d) Transepithelial electrical resistance (TEER) ratio measured 18 h after treatment with live *Parabacteroides* spp. (*n* = 5 per group). Data are expressed as fold change relative to the control (set as 1.0). (e) Expression levels of tight junction-related genes (*ZO-1*/*TJP1*, *CLDN4*, and *OCLN*) in Caco-2 cells after treatment with live *Parabacteroides* spp. (*n* = 5 per group). Expression was measured via quantitative real-time PCR and normalized against *GAPDH* expression. Statistical significance was assessed using Spearman’s correlation analysis (c) and Dunnett’s multiple comparisons test (d and e). *p < 0.05; **p < 0.01; ***p < 0.001. Pm ASVs, ASVs most closely related to *P. merdae*; Pd ASVs, ASVs most closely related to *P. distasonis*; Pj ASVs, ASVs most closely related to *P. johnsonii*; Pm + Pd + Pj ASVs, combined abundance of Pm ASVs, Pd ASVs, and Pj ASVs.

To identify the *Parabacteroides* lineages associated with the intestinal barrier integrity, BLASTn homology searches were conducted on ASVs assigned to the *Parabacteroides* genus against the NCBI 16S rRNA sequence database, followed by taxonomic annotation (Figure 3a; Table S4). Among the identified ASVs, those most closely related to *P. merdae* (Pm ASVs) constituted the most abundant lineages, followed by ASVs related to *P. distasonis* (Pd ASVs) and *P. johnsonii* (Pj ASVs), which together accounted for approximately 84% of the total relative abundance within the genus (Figure 3b). To assess the relationship between these lineages and the intestinal barrier integrity, Spearman’s correlation analysis was performed for zonulin levels and relative abundance of each lineage, as well as for their combined abundance. Only the combined relative abundance of Pm ASVs, Pd ASVs, and Pj ASVs exhibited a significant and negative correlation with zonulin levels (Figure 3c and Figure S4a), suggesting that these lineages may act collectively to influence the barrier function. To directly test their effects on the epithelial barrier integrity, the Caco-2 monolayer assay was performed. Compared with the control group, all three species significantly increased the TEER and upregulated the expression of tight junction-related genes, including *TJP1*, *CLDN4*, and *OCLN* (Figure 3d, e). In contrast, HK preparations of the three *Parabacteroides* species failed to produce significant changes in the TEER or in the expression of tight junction genes relative to the control (Figure S4b, c). Viability assays confirmed that bacterial counts in the coculture supernatants exceeded 10^7^ colony-forming unit (CFU)/mL for each species (Figure S4d), indicating that live bacteria were present throughout the experiment. These results indicated that *P. merdae*, *P. distasonis*, and *P. johnsonii* can enhance the intestinal barrier function *in vitro* and that this effect possibly requires the presence of viable bacteria.

### Fecal sialic acid correlates with *Parabacteroides* and promotes its growth

**Figure 4.**
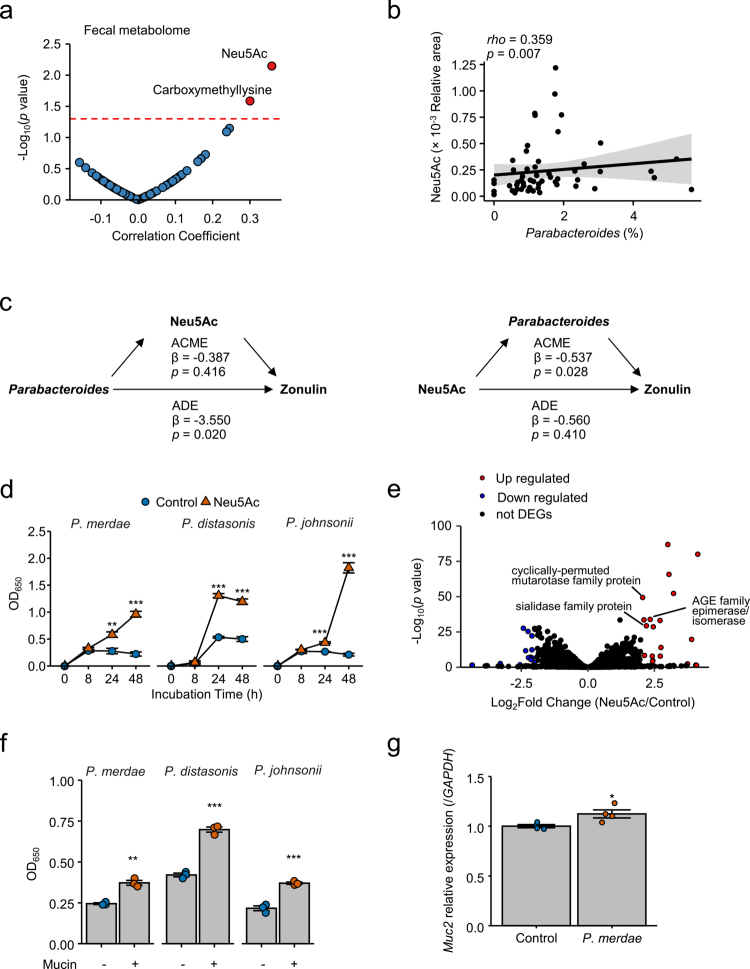
Intestinal factors influencing the *Parabacteroides* abundance. (a) Spearman’s correlation analysis between fecal metabolome and *Parabacteroides* abundance (*n* = 55). (b) Scatter plots showing correlations between fecal Neu5Ac levels and *Parabacteroides* abundance (*n* = 55). (c) Mediation analysis of the associations among *Parabacteroides*, Neu5Ac, and zonulin (*n* = 55). *Parabacteroides* abundance and Neu5Ac levels were log-transformed. Estimates (*β*) and *p* values of average causal mediation effects (ACME) and average direct effects (ADE) adjusted for age, sex, and body mass index (BMI) are shown. (d) Growth-promoting effects of Neu5Ac on *Parabacteroides* spp. in sugar-free YCFA medium (*n* = 3 per group). (e) Volcano plot of differentially expressed genes in *P. merdae* cultured with or without Neu5Ac supplementation (*n* = 3 per group). Significance threshold: |log_2_ fold change| > 2.0 and *p* < 0.05. (f) Growth-promoting effects of mucin on *Parabacteroides* spp. in sugar-free YCFA medium (*n* = 3 per group). (g) *Muc2* gene expression measured 24 h after treatment with live *P. merdae* (*n* = 4 per group). Statistical significance was assessed using Spearman’s correlation analysis (a and b), mediation analysis in the mediation package (c), Student’s *t*-test (d, f, g), and differential gene expression analysis in DESeq2 package (e). *p < 0.05; **p < 0.01; ***p < 0.001.

Fecal metabolites not only act as mediators of host–microbiota interactions but also as modulators of microbial growth and community composition.^34^ To identify metabolites associated with *Parabacteroides* abundance, we conducted correlation analyses between their relative abundance and the fecal metabolome. Among the detected compounds, two metabolites—Neu5Ac (sialic acid) and carboxymethyllysine—were significantly associated with *Parabacteroides*, with Neu5Ac showing the strongest positive correlation (Figure 4a, b; Table S5). To assess the robustness of the associations, RDA with stepwise variable selection was performed, followed by multiple linear regression using the retained variables, which identified three metabolites—Neu5Ac, trimethyllysine, and 2-aminobutyric acid—to be significantly associated with *Parabacteroides* (Neu5Ac: *β* = 0.159, 95% CI 0.045 to 0.272; trimethyllysine: *β* = 0.153, 95% CI 0.054 to 0.251; 2-aminobutyric acid: *β* = −0.186, 95% CI −0.357 to −0.015; R² = 0.26; Figure S5a). In an additional regression model adjusted for age, sex, and BMI, Neu5Ac levels remained significantly associated with *Parabacteroides* abundance (*β* = 0.151, 95% CI 0.025 to 0.277; R² = 0.11; Figure S5b). Among gut microbiota taxa, *Parabacteroides* exhibited the strongest positive correlation with Neu5Ac (Figure S5c; Table S6). Therefore, Neu5Ac was selected for further investigation in the context of barrier function. Mediation analysis revealed a significant indirect effect of Neu5Ac on zonulin through *Parabacteroides*, whereas the reverse pathway—from *Parabacteroides* to zonulin via Neu5Ac—was not significant (Figure 4c). Notably, Neu5Ac itself was not directly correlated with zonulin levels (Figure S5d) and did not affect TEER values in Caco-2 monolayer assays (Figure S5e)—consistent with the mediation results. Based on these observations, subsequent analyses focused on how Neu5Ac influences *Parabacteroides*. Supplementation of Neu5Ac to YCFA medium without sugars significantly enhanced the growth of *P. merdae*, *P. distasonis*, and *P. johnsonii* compared with that in no-sugar controls (Figure 4d). Based on these findings, the impact of *Parabacteroides* abundance on intestinal barrier function was further investigated. In a dose–response experiment using the most dominant lineage, *P. merdae*, in a Caco-2 monolayer assay, only the higher inoculum significantly increased TEER values compared with the controls (Figure S6). Additionally, transcriptomic analysis of *P. merdae* revealed Neu5Ac-associated gene expression changes, with 20 genes getting upregulated and 15 downregulated upon Neu5Ac exposure (Figure 4e, Table 2). The upregulated genes included those encoding sialidase family proteins, major facilitator superfamily (MFS) transporters, AGE family epimerases/isomerases, and cyclically permuted mutarotase family proteins—functions consistent with sialic acid catabolism and transport.^35^^,^^36^ Because sialic acid is typically located at the terminal ends of host-derived mucins,^35^^,^^36^ we next examined the utilization of mucins. All three *Parabacteroides* species showed significantly enhanced growth in YCFA medium without sugars supplemented with purified mucin compared with that in the no-mucin controls (Figure 4f). Furthermore, coculture of *P. merdae* with LS174T cells significantly increased the expression of *Muc2* (Figure 4g), indicating a reciprocal relationship in which *Parabacteroides* spp. utilize and potentially stimulate mucin production. These results indicate that the growth of *Parabacteroides* is strongly promoted by host-derived glycans such as mucin-associated sialic acids and that a sufficient abundance of viable bacteria may be required to enhance the intestinal barrier function.

**Table 2.** Differentially expressed genes of *P. merdae* by Neu5Ac.

gene	Log_2_FoldChange	*p* value	padj	gene products	change
NQ542_RS08110	3.007	1.220E-87	4.269E-84	multidrug efflux RND transporter permease subunit	Up regulated
NQ542_RS07680	4.132	1.000E-80	1.750E-77	hypothetical protein	Up regulated
NQ542_RS08105	3.050	1.780E-66	2.076E-63	efflux transporter outer membrane subunit	Up regulated
NQ542_RS14780	3.222	5.610E-53	4.907E-50	hypothetical protein	Up regulated
NQ542_RS14020	2.061	4.050E-50	2.834E-47	cyclically-permuted mutarotase family protein	Up regulated
NQ542_RS14000	2.344	1.280E-34	7.465E-32	AGE family epimerase/isomerase	Up regulated
NQ542_RS00075	2.111	2.850E-34	1.425E-31	hypothetical protein	Up regulated
NQ542_RS08115	2.715	8.040E-34	3.126E-31	efflux RND transporter periplasmic adapter subunit	Up regulated
NQ542_RS14010	2.202	4.270E-30	1.494E-27	sialidase family protein	Up regulated
NQ542_RS09140	2.461	2.220E-29	7.062E-27	outer membrane beta-barrel protein	Up regulated
NQ542_RS04120	3.902	1.950E-20	2.274E-18	hypothetical protein	Up regulated
NQ542_RS14005	2.750	1.020E-14	5.577E-13	MFS transporter	Up regulated
NQ542_RS01120	2.132	4.300E-09	6.657E-08	aminoacyl-tRNA hydrolase	Up regulated
NQ542_RS16850	2.724	1.380E-08	1.886E-07	hypothetical protein	Up regulated
NQ542_RS04010	2.422	1.380E-08	1.886E-07	tRNA-Thr	Up regulated
NQ542_RS12605	2.445	1.920E-05	1.454E-04	DNA repair protein RadC	Up regulated
NQ542_RS04005	2.436	5.200E-05	3.589E-04	tRNA-Tyr	Up regulated
NQ542_RS09395	3.749	0.004	0.016	hypothetical protein	Up regulated
NQ542_RS09930	4.060	0.026	0.078	phage integrase SAM-like domain-containing protein	Up regulated
NQ542_RS10315	4.087	0.042	0.114	DUF3945 domain-containing protein	Up regulated
NQ542_RS16000	−2.422	2.540E-28	6.837E-26	arginase	Down regulated
NQ542_RS13620	−2.246	4.200E-26	7.628E-24	TonB-dependent receptor	Down regulated
NQ542_RS15995	−2.098	5.620E-23	8.550E-21	ornithine--oxo-acid transaminase	Down regulated
NQ542_RS16755	−2.097	4.310E-13	1.714E-11	uncharacterized gene	Down regulated
NQ542_RS07620	−2.336	2.010E-12	6.828E-11	linear amide C-*N* hydrolase	Down regulated
NQ542_RS10795	−2.145	7.530E-08	9.023E-07	TonB-dependent receptor	Down regulated
NQ542_RS07340	−2.230	1.530E-07	1.721E-06	carboxypeptidase-like regulatory domain-containing protein	Down regulated
NQ542_RS14480	−2.096	2.480E-07	2.729E-06	TonB-dependent receptor	Down regulated
NQ542_RS17890	−2.135	3.450E-06	3.025E-05	uncharacterized gene	Down regulated
NQ542_RS07355	−2.004	3.560E-04	0.002	STN and carboxypeptidase regulatory-like domain-containing protein	Down regulated
NQ542_RS08975	-3.287	0.002	0.010	TraG family conjugative transposon ATPase	Down regulated
NQ542_RS16145	−2.549	0.018	0.058	F0F1 ATP synthase subunit epsilon	Down regulated
NQ542_RS07850	−2.029	0.022	0.067	hypothetical protein	Down regulated
NQ542_RS06580	−4.349	0.029	0.085	hypothetical protein	Down regulated
NQ542_RS08965	−4.339	0.029	0.086	DUF4141 domain-containing protein	Down regulated

## Discussion

In this study, we investigated the relationship between the intestinal barrier function, gut microbiota, and fecal metabolites in older adults to gain insight into factors that may help preserve the barrier integrity during aging. Our findings highlight *Parabacteroides* as a genus that enhances the intestinal barrier function, while host-derived substrates, such as sialic acid and mucin, promote their growth.

Consistent with previous studies on the intestinal barrier function and inflammation,^5^^,^^8^ our findings confirmed a significant association between the barrier integrity and inflammatory markers, which remained robust after adjustment for age, sex, and BMI. Earlier reports have linked factors, such as dietary fiber intake,^37^ GSRS,^38^ and sleep quality^39^ to the barrier function; however, these associations were not observed in our study. A plausible explanation lies in differences in study populations: previous studies often involved patient cohorts with already compromised barrier integrity, where external factors may exert stronger or more detectable effects. In contrast, our participants were relatively healthy older adults, in whom variability in the barrier function was likely narrower, making such associations more difficult to detect.

Among the gut microbiota, *Parabacteroides* emerged as the only genus consistently linked to the barrier marker, whereas well-known SCFA producers, such as *Bifidobacterium*, *Faecalibacterium*, and *Akkermansia*, which are often associated with barrier integrity via their metabolic activity,^14-19^^,^^37^^,^^40^ showed no such relationship. This suggests that, in older adults, mechanisms associated with *Parabacteroides* may be more relevant to maintaining the barrier function than the SCFA production itself, indicating that factors beyond SCFA generation could play an important role. Supporting this notion, fecal acetate and propionate did not exhibit any relationship with the barrier markers; however, butyrate was positively associated with zonulin levels. Given that higher zonulin levels reflect increased intestinal permeability, this association does not necessarily indicate a barrier-protective effect and may instead reflect context-specific influences, with other factors—potentially linked to *Parabacteroides*, such as aryl hydrocarbon receptor ligands or secondary bile acids—playing a more prominent role in maintaining the barrier integrity in this population. Consistent with this concept, the MaPLE trial demonstrated that a polyphenol-rich dietary intervention in older adults reduced serum zonulin levels, accompanied by changes in the gut microbiota and serum metabolome, thereby highlighting the role of microbe–metabolite interactions in regulating intestinal barrier function.^41^

Previous studies have linked certain *Parabacteroides* species to barrier enhancement in experimental animal models and cellular models;^42^^-^^46^ however, evidence from general populations is limited. Our findings in older adults indicate that this activity is not confined to disease contexts and may represent a broader ecological role of the genus. Identification of *P. merdae*, the most abundant lineage in our study, as an additional contributor expands the functional repertoire of *Parabacteroides* and indicates that barrier-supporting effects could be a conserved trait among multiple species. The possible requirement for viable cells points to active metabolic or signaling processes, consistent with the idea that select commensals dynamically interact with the epithelium to reinforce tight junctions and mucus production. In the context of aging, such mechanisms may help preserve mucosal integrity and could be leveraged in dietary or probiotic strategies to counteract the decline of barrier function.

Fecal metabolomics, which reflects both microbial metabolism and host- or environment-derived factors shaping the gut ecosystem,^34^ identified Neu5Ac as the metabolite most strongly associated with the *Parabacteroides* abundance. As a terminal component of mucin glycans, sialic acid provides a competitive nutrient niche exploited by mucin-degrading taxa such as *A. muciniphila.*^15^ Our data suggest that *Parabacteroides* occupies a similar niche, as evidenced by the upregulation of sialic acid catabolism genes and enhanced growth in the presence of sialic acid or mucin *in vitro*. The concurrent stimulation of *Muc2* expression implies a feedback loop in which *Parabacteroides* promotes its own resource base, potentially stabilizing its population under fluctuating dietary conditions. Such a strategy, paralleling that in *A. muciniphila,*^47^^,^^48^ may explain the persistence and barrier-supportive role of *Parabacteroides* in the aging gut.

This study has some limitations. First, the relatively small sample size may limit the generalizability of the findings. Moreover, the observed range of zonulin levels was relatively narrow and did not indicate overt intestinal barrier dysfunction, potentially constraining the interpretability of associations across a broader spectrum of barrier integrity. Future studies with larger and more heterogeneous populations are therefore warranted.

Second, although zonulin and LBP are commonly used serum biomarkers of intestinal barrier function, both have inherent limitations. Commercial zonulin elizas have been reported to cross-react with proteins other than pre-haptoglobin 2,^49^ raising concerns about assay specificity. LBP, on the contrary, reflects systemic exposure to bacterial lipopolysaccharide rather than direct epithelial permeability. Thus, reliance on these markers alone provides an incomplete assessment of intestinal barrier status. Future studies incorporating additional metrics, such as plasma LPS or urinary sugar permeability assays, are needed to provide more robust and multidimensional validation.

Third, the cross-sectional nature of the study precludes any inference of causality among intestinal barrier function, gut microbial composition, and associated fecal metabolites. Longitudinal and interventional studies will be required to establish causal relationships.

Finally, although *Parabacteroides* spp. were significantly associated with barrier-related markers, the *in vitro* experiments were conducted using static Caco-2 monolayer cultures, which do not completely recapitulate the complex, dynamic environment of the human gut. These models lack critical physiological features, such as immune cell interactions, inflammatory signaling, and luminal flow, all of which may influence host–microbe dynamics *in vivo*; notably, interactions between heat-inactivated bacteria and immune cells may still occur *in vivo* and are not captured by static epithelial models. Advanced experimental systems, including gut-on-a-chip platforms,^50^ may offer greater physiological relevance and help elucidate the mechanistic role of *Parabacteroides* in maintaining intestinal integrity.

In conclusion, in older adults, *Parabacteroides* was significantly associated with the intestinal barrier function, and fecal sialic acid appeared to support their proliferation. These findings identify *Parabacteroides* as a promising target for interventions to preserve barrier integrity and reduce age-related inflammation, warranting future studies to determine whether modulating the abundance of this genus can promote healthy aging.

## Supplementary Material

Supplementary Material Supplementary Figures

## Data Availability

Data are available from the corresponding author upon reasonable request. The raw sequence data generated in this study have been deposited in the DNA Databank of Japan (DDBJ) Sequence Read Archive under the BioProject numbers PRJDB37714.
