# Supplementary material for: Sialic acid-responsive Parabacteroides is linked to gut barrier integrity in older adults
Source: Gut Microbes. 2026 Feb 10;18(1):2627093. doi: 10.1080/19490976.2026.2627093 (PMC12893687; doi:10.1080/19490976.2026.2627093)
Supplement: Supplementary Material — Supplementary Figures [file KGMI_A_2627093_SM6467.docx]

**Supplementary Figures**

**Figure S1. Relationships among gut barrier biomarkers and their associations with categorical and continuous variables from phenotypic metadata (*n* = 55).**

1. Spearman’s correlation analysis between zonulin and lipopolysaccharide-binding protein (LBP) levels.
2. Multiple linear regression analysis of zonulin with log-transformed blood glucose, triglyceride, and Chalder fatigue scale (CFS) adjusted for age, sex, and body mass index (BMI).
3. Comparison of zonulin and LBP levels in categorical variables.

Exercise, regular exercise habit (Yes/No); Alcohol, Alcohol intake habit (Yes/No); Smoking, current smoking status (Yes/No). Statistical significance was assessed using Spearman’s correlation analysis (a), the Wilcoxon rank-sum test (b), and multiple linear regression analysis (c).

**Figure S2. Additional analytical approaches examining associations between　intestinal barrier markers and gut microbiota (*n* = 55).**

1. Redundancy analysis (RDA) examining associations between gut microbiota taxa and zonulin using stepwise variable selection based on adjusted R². Gut microbiota abundance data were log-transformed.
2. Differential abundance analysis of gut microbiota taxa associated with zonulin and lipopolysaccharide-binding protein (LBP), using the DESeq2 package.

**Figure S3. Spearman’s correlation analysis of zonulin and lipopolysaccharide-binding protein (LBP) levels with acetate, propionate, and butyrate levels (*n* = 55).**

**p* < 0.05; ***p* < 0.01.

**Figure S4. ASV-level associations of *Parabacteroides* with zonulin and assessment of heat-killed (HK) *Parabacteroides* spp. on intestinal barrier function in Caco-2 cells.**

1. Scatter plots showing correlations between zonulin levels and abundance of each *Parabacteroides* lineage or their combined abundance (*n* = 55).
2. Transepithelial electrical resistance (TEER) ratio measured 18 h after treatment with heat-killed (HK) *Parabacteroides* spp. (*n* = 5 per group). Data are shown as relative values normalized against the control group (set as 1.0).
3. Expression levels of tight junction-related genes (*ZO-1/TJP1*, *CLDN4*, and *OCLN*) in Caco-2 cells treated with HK *Parabacteroides* spp. (*n* = 5 per group). Gene expression was quantified via real-time PCR, normalized against *GAPDH* expression.
4. Culture medium from the coculture of *Parabacteroides* spp. and Caco-2 cells was directly plated onto Gifu Anaerobic Medium (GAM) agar and incubated for 4 days at 37°C in an anaerobic chamber. Colony-forming units (CFUs) were counted and reported as log_10_ CFU/mL (*n* = 4 per group).

HK *Parabacteroides* spp. were prepared by heating bacterial suspensions at 90°C for 15 min. Statistical significance was assessed using Dunnett’s multiple comparisons test (b and c). N.S., not significant. Pm ASVs, ASVs most closely related to *P. merdae*; Pd ASVs, ASVs most closely related to *P. distasonis*; Pj ASVs, ASVs most closely related to *P. johnsonii*; Pm+Pd+Pj ASVs, combination of Pm ASVs, Pd ASVs, and Pj ASVs.

**Figure S5. Relationship among *Parabacteroides*, fecal N-acetylneuraminic acid (Neu5Ac), zonulin, and intestinal barrier integrity.**

1. Multiple linear regression analyses of *Parabacteroides* with fecal metabolites selected by redundancy analysis (RDA) using stepwise variable selection (*n* = 55). Fecal metabolites were log-transformed.
2. Multiple linear regression analysis of *Parabacteroides* with Neu5Ac adjusted for age, sex, and body mass index (BMI) (*n* = 55). *Parabacteroides* abundance and Neu5Ac levels were log-transformed.
3. Heatmap indicating Spearman’s correlation coefficients between Neu5Ac levels and gut microbiota taxa. Only genera with the average relative abundance of at least 1% were included (*n* = 55).
4. Scatter plots showing correlations between zonulin levels and Neu5Ac levels (*n* = 55).
5. Transepithelial electrical resistance (TEER) ratio after 18 h of Neu5Ac treatment in Caco-2 cells (*n* = 4 per group). Data are shown as fold change relative to the control (set as 1.0).

Statistical significance was assessed using multiple linear regression analysis (a and b), Spearman’s correlation analysis (c and d), and Dunnett’s multiple comparisons test (e). **p* < 0.05; ***p* < 0.01; ****p* < 0.001; N.S., not significant.

**Figure S6. Transepithelial electrical resistance (TEER) ratio after 18 h of exposure to different doses of live** ***P. merdae* in Caco-2 cells (*n* = 4 per group).**

Data are shown as fold change relative to the control (set as 1.0). Statistical significance was assessed using Dunn’s test following a Kruskal–Wallis test. **p* < 0.05.
